# Supplementary material for: Are carbon emissions trading and green financial instruments synergistic? -Comprehensive quantitative research based on content analysis
Source: PLoS One. 2024 Mar 7;19(3):e0298601. doi: 10.1371/journal.pone.0298601 (PMC10919601; doi:10.1371/journal.pone.0298601)
Supplement: S1 Data — (ZIP) [file pone.0298601.s001.zip › Data/地级市Z_F工作报告.zip!/─┌├╔╣┼/░n═╖╩╨/└┤╫╘╣1⁄2╓┌║┼í╛┬φ┐╦╩2╛▌═°í┐_2017.docx]

各位代表：

现在，我代表市人民政府向大会报告工作，请予审议，并请市政协委员和列席会议的同志提出意见。

一、2016年工作回顾

过去一年，在市委的坚强领导下，全市上下深入学习贯彻习近平总书记系列重要讲话和考察内蒙古重要讲话精神，坚决落实中央、自治区和市委决策部署，在抢抓机遇中干事创业，在攻坚克难中砥砺前行，奋力开创“十三五”发展新局面。全市地区生产总值3867.6亿元，增长7.6%；固定资产投资2955.8亿元，增长14.4%；社会消费品零售总额1400.2亿元，增长9.7%；一般公共预算收入271.2亿元，增长7.5%；城乡居民收入分别达到40955元和14692元，均增长7.5%；城镇登记失业率控制在3.89%。

（一）经济发展稳中提效

全力稳定经济增长。充分发挥工业支撑作用，制定实施稳工业、助企业的一系列政策措施，为企业争取上级各类扶持资金24.7亿元，协调落实银行贷款830亿元。全年规模以上工业企业增加值增长8.9%，位居全区前列；企业效益明显好转，利润总额增长261.4%。充分发挥投资关键作用，实施亿元以上重点项目831个，完成投资2586.7亿元，400个项目陆续建成，实施项目和竣工数量位居全区首位。承办自治区首届蒙商大会，积极参与内蒙古大数据产业推介会等招商引资活动，引进国内区外到位资金762.7亿元，增长15.7%。充分发挥消费基础作用，大力改善消费供给和市场流通，全市开办电商业务企业达到1600多家，进入国家电子商务示范城市创建行列。举办第26届全国书博会等61场大型展会，参展人数达到330万人次，成交额近600亿元。充分发挥金融保障作用，支持企业直接融资473.6亿元。拓展政府融资平台功能，策划设立总规模805.9亿元的产业基金22个，总规模368.5亿元的12个基金获批。全社会融资总额达到3986.6亿元，超过金融机构存款余额732.8亿元，有力支持了企业发展和项目建设。

扎实推进“三去一降一补”。拆除包钢2号高炉和宝鑫特钢高炉，化解钢铁过剩产能193万吨。有效推动去库存，全年商品房销售套数、面积分别增长39.3%和24%。改善地区金融环境，金融机构不良贷款率2.28%，低于全区1.53个百分点，保持在合理区间。打好减免收费、电价补贴等政策“组合拳”，营改增改革为企业减税15亿元以上，促进规模以上工业企业主营业务收入成本下降1%。加大基础设施、科技创新、生态环境、社会民生事业等方面投入，短板领域得到有效加强。

（二）转型升级步伐加快

大力改造提升传统产业，新增汽车用钢、高强耐磨贝氏体钢轨等161种高附加值产品，新增40万吨铝深加工产能，完成400台传统机床数字化改造。大力培育战略性新兴产业，高端装备制造业增加值增长11.2%，稀土功能材料产量增长19.8%，风光清洁能源装机容量分别达到376万千瓦和60万千瓦，增长7.5倍和1.6倍，全市战略性新兴产业增加值占GDP比重达到7%。大力发展现代服务业，服务业投资占比和经济总量占比均超过50%，三次产业结构优化为2.5:47.1:50.4。公路港、铁路港、航空港建设扎实推进。旅游总收入突破400亿元，增长24.1%。福强物业、新达科技、惠民水务、塞北机械等10户企业在新三板挂牌，在区域性股权交易中心挂牌企业达到705户。民生银行、内蒙古文化产权交易所落户包头，包银消费金融公司获准开业，新时代信托扩增资本60亿元，组建村镇银行和农商行工作取得积极进展。大力发展现代农牧业，引进中牧、中粮、中农批等一批国内知名企业，现代农牧业园区和食品加工园区入驻企业分别达到112家和52家，新增自治区级龙头企业13家、著名知名商标28个，农畜产品加工转化率达到63%。包头进入国家农产品质量安全追溯试点城市行列，获评“中国羊肉美食之都”。大力实施科技创新五大工程。33项重点科技项目、32项产学研项目和44项产业化项目全面推进，38项成果转化落地，发布18项稀土产品标准，农牧业科技贡献率达到57%。新增20家国家级高新技术企业，20家市级创新型试点企业、10家科技小巨人企业。与科研院校合作领域不断拓展，自治区首家石墨烯材料研究院落户包头，创建国家军民融合创新示范区迈出坚实步伐。全年R&D经费支出达到65亿元，增长21%，我市成为国家促进科技与金融结合试点城市。制定科技人才扶持政策，实施大学生集聚计划，8家众创空间晋升国家级。智慧城市建设扎实推进，获批“宽带中国”试点城市。

（三）改革开放深入推进

抓好重点领域改革攻坚。制定公布政府权责清单、投资领域负面清单和公共服务事项清单。行政审批要件精简20%以上，建成覆盖全市的“网上政务大厅”和“掌上政务大厅”。商事登记实施“六证合一、一照一码”和个体工商户“两证整合”，市场监管“双随机、一公开”机制不断强化，全年新登记注册企业增长13.1%。深化国资国企改革，启动实施公益类企业成本规制试点。实施混合所有制项目20个，带动投资148亿元。铝业园区配售电改革试点获得国家批准。不动产统一登记发证全面启动。完善龙头企业与农牧民利益联结机制，产业化经营参与率达到70%。土地草场确权工作扎实推进，基本农田红线、基本草原划定和小型水利工程管理体制改革全面完成。各领域改革均取得阶段性成果。

主动参与区域协同发展。制定呼包鄂地区协同发展规划和实施方案，推动包银高铁、包西高铁列入国家“十三五”规划和中长期铁路网规划，包头成为国家规划的50个铁路重要枢纽城市之一。城市立体综合交通枢纽等重大工程全面开工建设，南绕城一级公路和包头环城铁路建成通车，包白快速列车实现对开，基础设施加速内连外通。举办第四次呼包银榆市长联席会议，提升了包头区域经济影响力。

强力推动对外开放。满都拉口岸成为国家进口肉类指定口岸，获批建设中蒙互市贸易区，包满铁路三期主体工程完工，口岸过货量达到80万吨，超过前三年总和。制定外贸扶持政策，开通包头至中亚国际集装箱班列，新增外贸企业124家、境外投资企业11家，在全国全区外贸持续下滑的背景下，我市进出口贸易总额完成17.2亿美元，增长10.9%。全面完成包头机场国际航站楼改造，国际航空口岸实现临时开放，飞往蒙古国、泰国、香港的国际和地区航线正式通航。在蒙古国成功举办包头特色商品博览会，与香港的经贸合作不断深化。中德党际第四次可持续发展对话走进包头，与澳大利亚、新西兰建立城际友好关系，对外开放空间加速拓展。

（四）城乡建设展现新貌

坚持规划先行、建管并重，实施城建项目224个，完成投资618亿元。北梁腾空区18个重点项目全面启动，新都市区开发建设扎实推进。全市新建改造45条道路和4座桥梁，三年打通80条断头路任务全面完成，累计建成地下综合管廊18.1公里。轨道交通规划获批，地铁控制中心开工建设。全市增设公共停车位32189个，添置新能源公交车460台，公交站点500米覆盖率达到80%。新建扩建14个公园广场，建成区绿化覆盖率达到44%，提高1.4个百分点。对200栋建筑进行了夜景亮化。城市执法体制改革全面开展，创建景观示范街、治理背街小巷和机动三轮车违法营运取得显著成效。全面完成“十个全覆盖”三年建设任务，改造危房5.2万户，硬化街巷5185公里，解决了29.3万人的安全饮水问题，全市96%的行政村通油路，1725个村庄旧貌换新颜，48.4万群众受益。

（五）生态环保继续加强

实施国家林业重点工程53.5万亩，重点区域绿化4.6万亩，新增果品经济林2万亩。组织开展打击破坏生态资源违法行为专项行动，大青山矿山治理工程全面启动。围封禁牧取得阶段性成果。四道沙河、二道沙河河道治理及东河河湖连通工程扎实推进，包头黄河国家湿地公园通过验收。实施环保项目112个，完成投资21.9亿元。加强环保监管执法，累计查处企业环境违法行为612起，对中央环保督察组交办问题进行了集中整改。全市空气质量优良天数达到269天，比2015年增加20天。单位GDP能耗下降4.92%，完成主要污染物减排任务。

（六）人民生活持续改善

财政民生支出占比达到82.2%。加大脱贫攻坚力度，贫困人口减少2万人。建成创业园孵化基地57个，稀土谷创业园成为国家级示范基地。发放创业担保贷款4.5亿元，占自治区的三分之一。城镇新增就业4.23万人，高校毕业生就业2.27万人，农牧民转移就业23.28万人。采取“一制一档”模式率先实现城乡医保并轨。为33户企业发放援企稳岗社保补贴3.26亿元，惠及职工近11万人。加强妇女儿童和残疾人工作，在自治区率先向困难失能老人发放护理补贴，向失独残独困难家庭发放一次性扶助金，残疾人康复中心和民政福利园区开工建设。全年改造棚户区28705套，整治老旧小区194个、538万平方米。深入推进平安包头建设，依法打击各类违法犯罪活动，强力整治社会治安突出问题，提升了群众安全感。加大安全生产监管力度，生产安全事故和死亡人数分别下降61.1%和11.5%。

（七）社会事业不断进步

新改扩建中小学、幼儿园45所，九原区和土右旗通过国家县域义务教育均衡发展评估验收。成功创建国家公共文化服务体系示范区，文艺精品不断推出，电视剧《安居》在央视一套黄金时间播出，“交响音乐季”等品牌文化活动让越来越多的市民走进高雅艺术殿堂。33家公立医院取消药品加成，包钢医院等7个医联体试点全面启动，家庭医生签约服务达到80.4万人。深入推进足球改革试点工作，成功举办多项国家、自治区级赛事。乡镇全民健身中心和社区、嘎查村健身路径等体育设施不断完善，群众体育文化活动蓬勃开展。全国质量强市示范市创建持续深化，食品药品安全监管不断加强。我市第四次获评全国法治宣传教育先进城市，蝉联国家卫生城市荣誉称号，入选国家健康城市建设试点。民族宗教工作开创新局面，创建全国双拥模范城实现“八连冠”。统计、人防、防灾救灾、红十字、外事侨务、文史档案等事业发展取得新成绩，税务、海关、检验检疫、气象等部门工作迈上新台阶，金融、保险、证券等机构为全市经济社会发展作出新贡献。

过去一年，我们努力加强政府自身建设，认真开展“两学一做”学习教育，严格遵守党规党纪，坚持不懈改进作风。主动自觉接受人大法律监督、工作监督和政协民主监督，广泛接受人民群众和社会监督。高度重视人大代表建议和政协提案办理工作，共办理人大代表建议141件、政协提案306件，办结率100%，对人大、政协调研报告和审议意见进行专门研究落实。依法全面履行政府职能，规范各项工作制度，大力推进政务公开和信息化建设，加强行政监察和审计监督，狠抓督查落实，政府执行力和公信力进一步提升。各级政府和政府工作部门抢抓机遇，一件一件事情盯，一步一个脚印走，一天赶着一天干，率先发展的积极性和主动性日益增强，不甘落后、真抓实干、创新创业的激情不断凝聚，为做好今后改革发展稳定各项工作奠定了坚实基础。

各位代表！过去一年取得的成绩，是市委坚强领导、统筹推进的结果，是市人大和市政协认真监督、大力支持的结果，是社会各界和有关方面热情参与、鼎力相助的结果，是全市广大干部群众苦干实干、顽强拼搏的结果。我代表市人民政府，向所有为包头建设与发展作出贡献的同志们、朋友们，表示衷心的感谢，致以崇高的敬意！

在总结成绩的同时，我们也清醒地认识到，包头发展还面临着不少困难和挑战。从外部环境上看，经济下行压力依然较大，市场预期不稳定，民间投资增长动力明显不足；经济发展新常态下的城市间竞争日趋激烈，我市传统优势正在减弱，科技创新成果转化能力有待提高，现代服务业和战略性新兴产业发展不足，经济发展的新动能还没有全面形成。从内部支撑上看，一些企业生产经营中存在的困难问题还没有得到有效解决，民营经济发展的环境仍待优化；对外开放整体水平不高、外资利用规模不大；财政收支矛盾加剧，旗县区发展不平衡；城乡建设管理有待加强，生态环境保护还需持续加大力度；居民收入增长放缓，农牧民收入与预期目标还有一定差距；脱贫攻坚、棚户区改造等任务十分艰巨，人民群众关心的空气质量、食品安全等热点问题还没有得到根本解决。从政府工作上看，对标先进城市，我们推动工作谋发展还有不小差距，研究政策、捕捉机遇的能力仍显不足，一些干部敢于比拼、争名进位的意识还需不断强化。对此，我们一定直面问题、解决问题，尽心竭力推动改革发展，决不辜负全市人民的重托。

二、2017年工作安排

今年政府工作的总体要求是：全面贯彻落实党的十八大和十八届三中、四中、五中、六中全会精神、习近平总书记系列重要讲话和考察内蒙古重要讲话精神以及治国理政新理念新思想新战略，按照中央经济工作会议、自治区党委十届二次全会和市委十二届二次全会的部署要求，坚持稳中求进工作总基调，以提高发展质量效益为中心，以推进供给侧结构性改革为主线，以提档升级、争创一流为目标，守住发展、生态、民生底线，强化创新驱动、投资拉动、项目带动，努力在增强发展后劲、优化工业结构、提升城市品位、改善人民生活四个方面实现新突破，在作风转变上下工夫、在工作落实上求实效，打造全区经济社会发展火车头、改革开放领头羊、统筹城乡发展先行区、生态环境保护示范区，挺钢铁脊梁、振包头雄风，以优异成绩迎接自治区成立70周年和党的十九大胜利召开。

今年主要预期目标是：地区生产总值增长8%左右，固定资产投资增长15%左右，社会消费品零售总额增长10%左右，一般公共预算收入增长6%左右，城乡居民人均可支配收入分别增长8%、8.5%左右。城镇登记失业率控制在3.9%以内。全面完成自治区下达的节能减排约束性指标。

实现上述目标，要重点做好以下几个方面工作。

（一）增投资上项目，全力推动综合经济实力实现新跨越。今天的项目就是明天的生产力。我们要全面开展“项目建设攻坚年”活动，组织实施总投资7737亿元的957个重点项目，年内完成投资2600亿元以上，竣工400个以上，为经济社会发展注入强大动力，助推率先发展。

围绕重大项目抓好“七网”建设。根据产业发展与项目布局，加快制定完善“七网”专项规划，实施总投资4363亿元的211个项目。铁路网总投资252亿元，开工建设包头至银川高速铁路、土右新型工业园区铁路和包头铝业古城湾铁路专用线，建成包满铁路三期和固阳明安至金山工业园区铁路，完成环城铁路电气化改造和土右嘉华铁路扩能改造，积极开展包西高速铁路（包榆段）前期工作，谋划推动呼包高铁项目。全面建成公铁空立体综合交通枢纽主体工程，争取以综合交通枢纽为包头高铁起点站，构建包西、包银“十字形”高铁通道。公路网总投资446亿元，全面建设白云鄂博至固阳、固阳至东河、白云鄂博至百灵庙3条高速公路，在全区率先实现县县通高速目标。开工建设托县至东河、满都拉口岸至白云、包头北绕城等一级公路，建成固阳至武川、希拉穆仁至百灵庙和边防公路。实施包茂高速公路包头至东胜段改扩建工程，启动跨黄河大桥通道项目。全面建成586公里旅游公路网，实现主要景区景点全覆盖。建设通村油路294公里，实现所有行政村通油路。航空网总投资16亿元，建成石拐、达茂通用机场，组建包头航空公司，推动包头机场升级为国际机场。优化国内航线，增加与省会城市的通航数量，开通俄罗斯、澳门等更多国际和地区航线，提升航空运力和服务水平。水利网总投资276亿元，全力推进城市水生态提升综合利用项目，基本完成昆河、四道沙河、二道沙河、东河“四纵”贯通工程。加快推动山北输水工程，努力解决区域性缺水问题。新增城区管道直饮水覆盖人口6万人，实施农村牧区饮水安全巩固提升工程，提高城乡居民饮用水质量。市政网总投资593亿元，开工建设地铁1号线、2号线一期工程，新建改建沼园南路等65条道路，建成新都市区、北梁新区和110国道、沼南大道等33公里地下综合管廊。启动环城北干线、昆北集中供热干线等工程，建设供热管网74公里。新建扩建污水处理厂，加快污水汇集管网与中水利用管网建设，千方百计提高污水处理率与中水利用率，解决城市既缺水又浪费水资源的矛盾，减少地下水开采量。大力推进陕京四线入包等天然气管道项目建设，新增天然气供给能力10亿立方米。能源网总投资2746亿元，新建昆区莫尼、新都市区世纪220千伏变电站和东河前明、昆区新城、石拐喜桂图、北梁北滨等一批110千伏变电站，提高城乡供电保障能力。加快实施包铝热电联产机组扩建和固阳金山工业园区电力及配套输电工程。推动包头北至华中特高压电力外送通道尽快获得国家批复。信息通信网总投资34亿元，加快建设大数据、云计算三期等项目。推进新建城区光纤网络和高速宽带网络全覆盖，加强公路沿线、旅游景区、农村牧区光纤通讯网络和无线网建设，新建基站1200座、光缆2840公里，骨干网出口带宽达到850G，80%的行政村实现光纤到村，不让农牧民在信息化进程中掉队，为建成西部地区信息化强市奠定坚实基础。

围绕重大项目全力招商引资。树立全市“一盘棋”思想，在招商引资上算大账、谋长远，制定出台更有竞争力和吸引力的招商引资优惠政策。鼓励各地区发挥自身优势，量身订制错位招商的配套政策。健全招商引资责任制，加强队伍建设，落实奖惩措施，营造地区、部门主要领导亲力亲为，专业队伍全方位、全过程、全身心跟踪服务的浓厚氛围。注重项目谋划，加强政策研究和工作对接，研判市场走势，跟进项目资金信息，重点围绕优势资源深度转化、特色产业延伸链条、应用科技创新合作、新型消费热点培育等领域开展精准招商。创新招商方式，充分运用以商招商、展会招商、网络招商等多种方式，加强与国内外大院大所、行业龙头企业洽谈对接，提高招商精准度和成功率。全年引进项目500个以上，引进国内区外到位资金增长15%以上。

围绕重大项目强化融资保障。抢抓政策市场机遇，大力推行PPP项目合作模式，激发民间投资活力，力争民间投资比重达到65%以上。积极发展政府参股的融资性担保公司，提高项目融资担保能力。精准投放工业、稀土、农牧业产业化、科技金融创新等产业基金，与银行、保险、基金等机构深度合作，扩大城市发展、棚户区改造等产业基金规模，以市场化运作模式助力项目建设。加快发展多层次资本市场，积极推动企业市场化债转股，推进资产证券化，支持企业上市融资和债券融资。拓宽融资渠道，加快组建地方金融资产管理公司、保险公司，设立区域性产权和金融资产交易平台。做大包银消费金融公司，全力支持一机集团成立内蒙古金融租赁公司。大力支持金融业创新发展，做大做强包商银行等地方金融机构。防范和化解企业债务、政府债务和金融风险，全力营造宽松安全的项目融资和金融发展环境。

（二）厚植发展优势，全力推动创新动能实现新集聚。深入实施创新驱动战略，围绕建设中西部地区乃至全国一流的创业创新高地，全面加快创新发展。

培育发展七大战略性新兴产业。编制《包头市战略性新型产业发展规划》及“七业”子规划和相应的三年行动方案，制定出台《加快战略性新兴产业发展的意见》，设立发展基金，加快建设战略性新兴产业基地，创建国家可持续发展议程创新示范区。加快建设以稀土为龙头的国家新材料产业基地。充分发挥我市稀土的比较优势与竞争优势，实施“稀土+”发展战略，理顺资源开发、运营、管理机制，支持北方稀土集团做大做强。加强稀土高新区建设，努力打造国内具有核心竞争力的一流创新型园区。发挥40亿元稀土产业基金和国家稀土转型支持资金引导作用，重点推动四大功能材料、稀土合金、铌钪钍综合利用等一批稀土和稀有金属转型升级项目，发展永磁电机、镍氢电池等终端产品，力争稀土功能材料产能达到10万吨，产量占到全国的1/5左右。积极创建国家稀土创新中心，发挥中科院包头稀土研发中心、包头稀土研究院等科研优势，新建一批稀土研发分支机构、技术转移中心和研发平台，力争实现磁材表面处理等3项重大技术突破，扩大镧、铈等元素的应用范围，完成无人机伺服电机等10项科技成果转化。强化稀土要素市场建设，依托包头稀土产品交易所，完善中国稀土价格指数，拓展稀土交易范围，重点推动本地稀土企业上线交易，着力打造中国稀土产品的交易中心、信息中心、投融资中心和定价中心。制定稀土产品标准，建设国家稀土产业计量中心，掌握稀土行业话语权。发挥包头自身优势，扩大稀土钢、稀土铝、稀土镁合金产业规模，延伸产业链，提高附加值。大力发展石墨烯新材料产业，重点实施核纯石墨、商用钠离子电池、高密度石墨烯及超级电容器等项目，拓展石墨烯新材料在冶金、核电、装备、环保行业的应用，打造国家石墨烯新材料制备及应用基地。

高端装备制造产业重点支持一机、北重、二冶、包铝等龙头企业在通用航空、智能制造等方面开发新产品，延伸产业链。推动工业化与信息化深度融合，加快建设工业大数据平台，打造一批数字车间、智能工厂。探索建立“网络协同制造联盟”，力争联网数控设备达到600台套，构建“互联网+工业制造”的创新发展模式，建设中西部地区网络制造基地。新能源产业重点推动采煤沉陷区光伏基地一期项目并网发电，年内启动二期工程。争取千万千瓦级风电基地通过国家批复并开工建设，全面推动一批光热和储能项目落地，力争产值实现50亿元。牢牢把握国家大力发展第三代核电、启动内陆核电站建设的政策机遇，加快建立核电燃料元件研发中心，进一步做大做强核电燃料元件产业。引进科研机构开发氢燃料和地热资源。支持杉杉集团与比亚迪集团扩大新能源汽车及组件生产规模。大数据云计算产业重点实施“互联网+”发展战略，成立专门机构，制定产业优惠政策，吸引更多的网络集团公司入驻包头。加快智慧教育、智慧医疗、智慧旅游、智慧管网等智慧包头专项领域的建设，推动数据资源共享，着力打造产值超百亿元的大数据产业集群。节能环保产业重点发展废旧金属资源再生利用、工业废弃物生产绿色建材、高效锅炉等产业项目，加大稀土在脱硫脱硝、DDS技术、节能照明等领域的推广应用。加快组建包头市生态节能环保产业公司，引进培育重点节能环保企业，创新节能环保服务模式，推动产业多元发展。生物科技产业重点支持东宝生物、华阳润生等企业，扩大明胶、胶原蛋白、磷酸氢钙等产品生产规模，推动赖氨酸、天然气和有机肥等生物科技项目发挥效能。大力引进基因生物等研发机构，夯实产业发展基础。蒙中医药产业重点发挥自身优势，加快发展甘草、黄芪、枸杞子等中草药产业。开展中蒙药材良种繁育和现代种养植、生产技术推广，启动建设蒙中医药产业园区，打造规范化种养植、规模化加工一体化基地。支持研发特色中蒙药材和相关新药、保健食品、茶饮、器械和洗化用品，满足健康消费需求。建设中蒙药材生产、流通及使用追溯体系，提高中蒙药产品质量和安全水平。

强化科技创新动力支撑。加大科技创新投入力度，健全完善多元投入机制，财政投入重点向科技创新倾斜，充分发挥30亿元科技创新基金作用，引进5家以上科技风险投资基金和天使投资基金，带动子基金规模达到10亿元，年内力争R&D经费支出增长25%。加大引才育才用才力度，完善相关人才政策，有针对性引进急需的高端研发人才、专业技术人才、创新创业人才，加强本地人才的培养和使用，激发人才活力，服务创新发展。设立人才发展基金，强化科技成果转化扶持和奖励。完善创新创业服务体系，加快建设一批创客空间、创新创业中心和新兴产业双创示范基地，营造激发创新动能的良好环境。加大创新平台建设力度，支持包钢、北重、中核北方建设国家重点实验室和国家工程技术研究中心，实施CAP1400先导组件研制、石墨烯可控制备等20项以上重大科技项目。与清华大学、北京航空航天大学、吉林大学、天津大学、中国农业大学等院校合作建设一批新型研发机构，在大数据、煤化工、机器人、新材料、农牧业科技等领域谋求突破。与澳大利亚莫纳什大学共建中澳（内蒙古）技术转移研究院，年内引进20项以上国际先进技术成果。建立本地高校、职业院校同企业协作服务平台，开展订单式专项科学研究和职业技术培训，构建科教融合、校企联合新模式。加大军民融合创新力度，用活用好科技评估和国防知识产权转化两个中心，以军民技术转化吸引产业集聚，打造军民融合产业园，加强军转民、民参军项目建设，全面创建国家军民融合创新示范区。加大质量标准化建设力度，开展质量提升行动，加快创建标准国际化创新型城市和全国质量强市示范城市。实施品牌强市战略，发展品牌经济，促进产品、服务和消费品牌有机结合，发挥商标对品牌的推动作用，落实“中国制造2025”，培育更多“百年企业”“百年老店”和“包头工匠”，打造包头品牌。

全力抓好工业园区建设。优化园区功能定位，引导工业园区根据产业定位做大做强主导产业和特色产业，构筑主业突出、特色鲜明、错位发展、协作配套的发展格局。提高园区承载能力，实施总投资27亿元的38个配套管网、污水处理等基础设施项目，年内所有园区建成区实现基础设施“八通一平”。大力推动产业向园区集中、企业入园发展，园区产业集中度达到82%。鼓励各旗县区至少与2家科研院校开展创新合作，力争在产业技术节点攻关上取得突破，建设创新型特色园区，把园区打造成为经济发展的主战场和聚宝盆。

（三）振兴实体经济，全力推动经济发展方式实现新转变。以市场为导向，打好传统产业提档升级攻坚战，实现转型发展。

加快改造提升传统产业。开展技术创新，加快产品和技术升级换代，实施总投资2520亿元的316个改造升级项目。钢铁产业重点调整产品结构、延伸产业链条，做精做优高附加值钢材产品，大力实施10万吨冷轧取向硅钢片、80万吨稀土现代铁素体不锈钢等项目，推动40万吨镀锌带钢及镀锌管等项目达产达效，力争钢铁产业实现产值930亿元。装备制造产业重点发挥龙头企业的集聚作用，带动相关配套企业形成完整的产业链条，实施光伏太阳能铸锭和金刚线切片机、现代煤化工装备、轨道交通装备制造等项目，提升传统装备制造业发展水平。有色金属产业重点推进160万吨铝合金材料、40万吨电解铜、3000吨高品质铝合金焊丝、30万吨铝镁合金、500万只铝轮毂等深加工项目，力争有色产业实现产值600亿元。煤化工产业重点实施60万吨乙二醇、煤焦油深加工等项目，年内力争神华70万吨煤制烯烃升级示范项目开工建设。

深入落实“三去一降一补”重点任务。大力支持企业发展，进一步降低企业融资、人工、物流、用地、用能、制度性交易成本和税费负担。在争取金融支持、引进险资、开拓市场、援企稳岗等方面全力以赴支持包钢等老工业企业转型发展。加快推动国有企业“三供一业”接收和剥离办社会职能，焕发老企业生机活力。实施中小企业成长工程，重点培育150户专精特新中小企业。推动钢铁、有色、装备制造、电力等企业开展协作配套，促进企业合作共赢。发挥政府采购引导功能，协调推动专用车辆、工程机械等优势产品进入区域公用项目采购市场，政府重点工程优先采用本地装备。全力保障生产要素供给，帮助企业拓宽产品销售渠道，提高工业产品销售率。支持居民合理住房需求，有效消化商品非住房库存，促进房地产企业健康发展。认真落实扶持民营经济发展的各项政策措施，坚持非禁即入、平等待遇，保障信息公开与对称，促进民营企业更好地参与公平竞争。建立与民营企业定期会商机制，在资本运作、技术攻关、人才引进等方面为民营企业排忧解难，依法保护民营企业家产权，让民营企业家更有尊严，在改革发展中有更多获得感。

做大做强服务业。推动生产性服务业向专业化和价值链高端延伸，生活性服务业向精细化高品质转变，促进产业结构调整和消费结构升级。物流方面，设立专项产业发展基金，实施好20个重点项目，完成投资55亿元。“公路港”年内建成投入运营，“铁路港”加快建设国际物流配送中心，建成B型保税物流中心，力争开通中东、中欧国际班列。“航空港”启动实施国际快件分拨中心项目，积极引进国内外知名第三方物流企业，加快建设国际空港物流园区。做大钢铁、有色、建材物流，发展绿色物流、冷链物流、城乡配送等新业态。积极发展总部经济，吸引资源、人才、信息、资金的集聚。电子商务方面，重点培育发展钢银电子交易平台、跨境电商平台、蒙西煤炭电子交易平台、大宗畜产品交易平台、稀土交易所、内蒙古文化产权交易所等电商平台，推动达茂、固阳创建国家级电子商务进农村示范县，全面建设国家电子商务示范城市。健康产业方面，设立健康产业基金，加快发展健康养老、保健用品、营养食品，重视和做好社区休闲健身、健康管理、健康咨询等工作，大力发展运动康复、保健器具、医疗器械等健康产业，打造健康产业基地。会展业方面，年内力争举办中国少数民族美食文化博览会、旅游用品博览会等大型展会60场以上，拉动消费600亿元以上，争取获批内蒙古会展产业示范园区。生活服务业方面，积极支持传统商贸流通企业和住宿餐饮企业利用先进信息技术实现线上线下深度合作，促进营销模式和服务方式创新。优化城市流通网络，着力打造一刻钟便民生活服务圈，实现便利店、便民菜店、代收代缴等基本便民服务全覆盖。推进法律咨询、会计税务等服务业走进生活服务领域，加快培育家政服务、中介服务等与群众生活密切相关的产业。突出抓好文化旅游融合发展，搭建文化旅游投融资平台，组建文化旅游集团公司，整合文化旅游资源。加快五当召5A级景区创建，集中力量打造九峰山、马鞍山、莲花山、希拉穆仁、南海、古长城遗迹等精品景区。启动建设军事旅游文化产业园、大青山红色旅游教育基地，开发包钢工业旅游等项目。加快打造沿黄休闲文化旅游带，建设具有蒙元文化特点的民族风情园。推动达茂、土右、石拐率先创建国家全域旅游示范区，敕勒川景区争创国家级旅游度假区，形成差异化、特色化文化旅游融合发展格局。办好中国民族旅游博览会、爱飞客飞行大会、中国游牧文化旅游节等文化旅游活动，打造特色品牌。开发冰雪、温泉、跨境旅游项目，发展冬季旅游、全时旅游、全民旅游，提升包头旅游地位，努力打造具有吸引力的知名旅游目的地，旅游人数和旅游收入增长15%以上。

加快发展“高精强特”现代农牧业。深入推进农牧业供给侧结构性改革，调整优化种养结构，稳步扩大菜薯乳肉四大主导产业规模，减少玉米播种面积，大力发展杂粮、汉麻、驴、梅花鹿等特色种养业。加快建设绿色农畜产品精深加工基地，与中牧、中农批等知名企业开展项目合作，现代农牧业园区培育引进企业50家以上，食品加工园区入驻企业超过60家，农畜产品加工转化率达到65%以上。大力培育龙头企业，新增国家级、自治区级龙头企业12家，市级以上示范合作社50家，力争82%的龙头企业与合作社及农牧户形成有效利益联结。加快建设农村牧区产权和使用权抵押融资平台，破解农牧民融资瓶颈，大力发展绿色加工、新型流通、旅游观光、健康养生等业态。狠抓农畜产品标准化生产，创建驰名、著名和知名商标45个，新增“三品一标”认证产品60个，扩大品牌市场占有率。健全质量安全监管体系，新建追溯点100个、投入品监管追溯试点100个，严把从农田牧场到餐桌的每一道防线。加快建设国家级番茄育种实验室和肉羊种羊繁育中心，积极创建国家农业高新技术产业示范区和国家农业可持续发展试验示范区。办好第18届中国绿色食品博览会、第4届中国·包头国际牛羊肉产业大会，实施绿色优质农畜产品进社区工程，拓宽销售渠道。

（四）增强发展活力，全力推动改革开放实现新突破。不改革开放就没有出路，我们要以更大的决心和更有力的举措，破除体制机制制约，以改革创新促进开放发展。

深化重点领域和关键环节改革。加大“放管服”改革力度，创建国家级社会管理和公共服务综合标准化试点，着力打造审批最快、收费最低、效率最高、服务最优的全国一流政务服务平台。加快工商注册“多证合一”，开展“减证便民”专项行动，让居民和企业少跑路、好办事、不添堵。完善事中事后监管，实现“双随机、一公开”监管全覆盖。加强信息共享、信用约束、联合惩戒、举报监督的配套衔接，强化社会信用体系建设。推进经营性国有资产集中统一监管，大力发展混合所有制经济，推动市属国有企业创新发展。全面推进铝业园区配售电改革试点。加快供销体制改革。规范发展各类社会组织，鼓励和支持承接政府购买服务项目，切实提高社会组织参与社会治理能力和服务水平。加强财源建设，严格财政预算管理，扩大绩效考评试点。深化行政执法体制改革，全面完成城市执法体制改革任务。健全完善编制、人事管理制度，深入落实职称制度改革。

加快发展外向型经济。坚定不移抓好对外开放，努力把包头打造成为“一带一路”的重要节点城市。全面加强满都拉口岸建设，建成进口肉类指定口岸，加快建设口岸互市贸易区和国际物流园区。积极推动赛音山达、塔奔陶乐盖至满都拉的2条公路和满都拉口岸至蒙古国珠恩巴音铁路项目。创新与俄蒙合作机制，组建内蒙古对外投资贸易集团公司，推动设立驻蒙、驻俄代表处。引导企业与蒙古国开展经贸交流，力争口岸过货量突破100万吨、出入境突破10万人次。推动无水港口岸实现手续办理“一站式”服务，支持小尾羊、鹿王等品牌企业建设海外基地、构建跨境营销网络，帮助有条件的企业开拓国际市场，进出口贸易总额增长20%。加强与欧美澳等发达国家和地区的全方位交流合作，加快培养和建设适应对外开放的外向型经济人才队伍，不断拓展开放广度和深度。

主动融入区域协同发展。制定呼包鄂协同发展年度实施计划，研究出台配套政策措施，推动建立会商合作机制，力争在交通、科技、旅游、网络通信等领域的合作取得实质性进展。与鄂尔多斯跨黄河共建自治区创新发展引领区，共同创建蒙西老工业城市和资源型城市产业转型升级示范区。认真落实呼包银榆经济区合作协议，积极对接京津冀协同发展，支持有条件的旗县区建设产业转移园区，在协同发展中争取主动，抢占先机。

（五）统筹城乡建设，全力推动文明和谐水平实现新提升。坚持走内涵发展道路，全面开展“城市建设管理提升年”活动，加快国家新型城镇化试点建设，促进协调发展。

加强城市规划设计。完善规委会决策机制，增强城乡规划的科学性和权威性。启动修编新一轮城市总体规划，协调推进“多规合一”，实现全市“一张图”管控，促进生态修复和城市修补。注重城市设计和单体建筑设计，落实城市建筑适用、经济、绿色、美观的方针，提升城市风貌特色。保护和修缮有历史价值建筑群落，传承历史记忆。加强城乡规划衔接和管控，旗县区实施重大工程、重大项目必须严格规划审批。规划建设全市劳务市场、农贸市场等专业市场。在城镇新区、公共建筑等领域大力推广钢结构和装配式建筑等新型建造方式。重视城市风道、河道、绿道的规划建设。

完善城市服务功能。实施总投资1976亿元的282个城建重点项目，年内完成投资800亿元以上。加快推进新都市区建设，重点抓好包医国际医院、城市展示与博物馆、文化艺术中心、地下空间开发等项目，促进产城融合和人口集聚，打造连接东西、贯通南北的新城市中心。加大北梁腾空区开发力度，加快实施包一中迁建等重点项目，配套引进总部经济、文化旅游等项目，打造全国棚户区改造规划建设、社会管理和可持续发展的典范。加快创建国家生态园林城市，新建改造兵工路带状公园、台地公园、召庙公园等30个公园，建成奥林匹克公园乒羽馆、网球馆、游泳馆及广场、水景瀑布等项目。加大小区绿化、单位绿化、街巷绿化力度，增加绿地214公顷。

切实提高城市管理水平。建立城市管理标准体系，打造市政设施维护基础数据共享平台和主城区数字化城管指挥系统。统一集中开展拆违建、拆临建、拆小锅炉、拆小煤炉和电厂超清洁改造大整治行动。新创建72条景观街，治理69条普通街巷，大幅提升市容环境质量。大力发展公共交通，增加新能源公交车，优化公交线路，方便群众出行。加强停车场、充电桩建设，继续治理机动三轮车违法运营，疏导解决机动车乱停乱放问题。投资4亿元实施环卫设施升级改造工程，提升环卫清扫机械化程度。依法治理居民小区私搭乱建、破墙开店、乱堆乱放、乱涂乱画等问题，加强物业监管，提高物业规范化服务水平。开展闲置土地清理工作，尽快解决房地产开发历史遗留问题，维护好城市开发建设秩序。

支持推动旗县区城镇建设。对旗县区城镇建设加强统筹协调，明确责任措施，强化项目和资金支持，加快培育特色小镇，完善基础设施和公共服务设施，促进农牧民就近城镇化。推动土右创建国家园林县城，固阳和达茂创建自治区园林县城。组织各旗县区全面推进棚户区和老旧小区改造，年内完成4.2万户棚户区和500万平方米老旧小区改造任务，做到改造标准不降低、工程实施要彻底。突出抓好北沙梁棚户区改造和环境综合治理，努力解决群众安居问题。支持山北地区协同发展，大力推动基础设施共建。加快推进美丽乡村建设，打造50个示范村和10个都市观光休闲农牧业示范基地，启动农村垃圾收集和污水处理工程，健全“十个全覆盖”管护长效机制，让农村成为安居乐业的美丽家园。

大力提升城市文明程度。实施优秀传统文化传承发展工程，深入挖掘和广泛宣传包头历史文化，擦亮城市品牌，讲好包头故事，树立包头自信。广泛开展家庭文明建设，让精神文明建设和先进文化更加贴近群众日常生活。加强文化基础设施建设，深入开展文化阵地服务、流动服务和数字文化服务，办好鹿城消夏文化节、民族音乐会、交响音乐季等活动，提高人民群众文化幸福指数。深化文化体制改革，加大文艺人才培养和引进力度。加强艺术精品创作生产规划和政策保障，围绕自治区成立70周年大庆和展示包头美好前景，打造一批文化艺术精品。挖掘民间文化艺术资源，关心和支持民间艺人创作，全面保护非物质文化遗产。制定扶持文化产业发展政策，设立专项基金，培育龙头企业，发展VR、AR、动漫等新业态和文化创意产业，推动文化产业增加值占GDP比重达到3.1%。持续推进文明城市建设，扎实做好全国文明城市迎检工作，实现“五连冠”目标。

（六）强化生态环保，全力推动生态环境质量实现新改善。发展决不能以牺牲环境为代价，我们要筑牢祖国北方重要的生态安全屏障，努力让包头的天更蓝、山更绿、水更清，实现绿色发展。

扎实推进生态建设工程。深入推进大青山生态修复工程，清理腾退保护区内工矿企业。实施国家重点林业工程40万亩以上，自治区重点区域绿化2万亩。大力发展林业产业，年内完成3万亩经济林建设任务。实施新一轮草原生态奖补政策，巩固围封禁牧成果。加强黄河国家湿地公园基础设施建设和生态保护修复。以支持生态建设、推动园林绿化、发展生态旅游等为重点，抓好100亿元生态产业基金设立运营，为建设美丽包头提供有力资金保障。

加强环保突出问题治理。全面打响大气污染治理攻坚战，投资22.7亿元实施37项大气污染综合治理工程，提升清洁生产水平，加强工程治理和管理，加快实现全市所有涉气企业达标排放。划定主城区高污染燃料禁燃区，全面推广清洁能源替代，综合施策、下大力气解决主城区燃煤散烧问题。强化秸杆焚烧、垃圾焚烧、露天烧烤和施工扬尘、道路扬尘、堆场扬尘等环境问题综合治理，加快淘汰黄标车和老旧车辆，进一步改善空气质量。全面加强水污染综合治理，严格执行“清源、活水、减负、节流、严管”五项措施，投资23.6亿元实施主城区西南区域污水治理等51项工程，加快创建国家节水型城市。全面开展土壤污染防治行动，投资2.5亿元实施42项土壤综合整治工程，建设全市土壤环境监测网络。加强环境监察执法规范化和标准化建设，严厉打击环境违法行为。认真对照中央环保督察组反馈意见，以铁的决心、铁的措施抓好整改，切实提高全市环境保护水平。

大力发展循环经济。以创建国家循环经济示范城市为抓手，打造“智慧能源”管控平台，加快建设包头铝业园区国家“城市矿产”示范基地、白云矿区矿产资源综合利用示范基地。全面推进国家节能减排财政政策综合示范市项目建设，切实提高资源利用水平。加快生活垃圾分类回收和再生资源回收“两网融合”，加强餐厨废弃物无害化处理和资源化综合利用。推进秸秆饲料化循环利用，变废为宝。

加快构建生态文明制度体系。严守生态保护红线，加强主体功能区建设，努力打造国家生态文明先行示范区。加快建立项目环评审批与规划环评、项目环境管理、区域环境质量联动挂钩机制，更好地发挥环评制度作用。严把土地预审和供应关，全面实施节地评价制度。加大国土资源执法监管力度，严控探矿权，严格探转采，严管采矿权。深入推进领导干部自然资源资产离任审计试点。建立覆盖全市所有固定污染源企业的排放许可制度，实现污染物排放有偿使用和交易。加快地区性碳排放交易市场建设步伐。

（七）增进民生福祉，全力推动人民生活水平实现新提高。坚持以人民为中心的发展思想，把民生小目标作为施政大方向，让人民群众拥有更多获得感，更好地体现共享发展。

坚决打赢脱贫攻坚战。扶贫脱贫是头号民生工程，必须按时保质保量完成。我们要坚持“六个精准”和“五个一批”基本方略，认真落实各项政策，加大资金投入、项目扶持和对口帮扶力度。坚持志智双扶，把着力点放在提高贫困群体自主发展能力上，充分调动贫困群众创造美好生活的积极性。严格实行脱贫工作责任制和贫困退出机制，切实解决边远地区、贫困地区“因病返贫”、“因病致贫”人群的医疗问题。年内实现所有国贫人口稳定脱贫，区贫县固阳县摘帽。

聚焦聚力增加居民收入。认真落实国家、自治区促进城乡居民增收的政策措施。抓好创业就业增收，全面开展“创业包头”行动，加大创业担保贷款扶持力度，新增5家国家和自治区级众创空间，力争创业培训8000人以上。建立覆盖城乡全体劳动者的技能培训制度，投入3000万元为3.5万人提供技能培训。认真做好复转军人、大学生、农牧民和就业困难人员的就业再就业工作，发展“互联网+家庭服务业”，开发更多就业岗位。全年新增城镇就业4.2万人，大学生就业2.4万人，农牧民转移就业23万人以上。抓好工资性增收，健全企业工资收入分配的正常增长和支付保障机制。完善机关事业单位津贴补贴和绩效工资制度，健全差别化激励机制。推动农村牧区三次产业融合发展，发展家庭手工业和庭院经济，支持龙头企业就近吸纳人口就业。积极稳妥推进土地草牧场确权颁证登记，加快流转交易市场建设，鼓励农村牧区土地入市流转交易。搭建民间投资信息平台和闲置资产交易平台，为增加居民财产性收入创造条件。

织密扎牢社会保障网。全面开展全民参保登记工作，加大社会保险扩面清欠力度，多措并举增加社会保险费收入，及时调整养老、医疗、失业、工伤等各项社会保险待遇水平。实施医保单病种付费方式改革，控制医药费用不合理增长。完善社会救助体系，提高低保、三无、五保、孤儿、一二级重度残疾人保障标准，进一步加强对困难群众的基本生活保障。加快建设残疾人康复中心和民政福利园区一期工程。大力发展养老事业和养老产业，加强居家养老服务平台、日间照料中心、互助幸福院、养老院等养老设施建设。重视做好老龄事业发展和老干部工作，扩大高龄补贴覆盖面，建成老年人教育发展中心，让老年人生活得更加安心、暖心、舒心。

办好人民满意的教育。坚持教育优先发展战略，全面实施教育提升三年行动计划。加大教育投入，新改扩建中小学、幼儿园20所，建设包头市特殊教育中心。推动昆区通过国家义务教育均衡县验收，组织开展城乡学校结对共建，提高优质教育资源共享水平。深入实施“领航校长”“卓越教师”培养计划和乡村教师支持计划，全面加强师资培训。建立健全教育教学质量考评体系，对成绩优秀的学校、校长、班主任、任课教师给予重奖，激发广大教师从教热情。围绕产业、项目，大力发展定向委托职业教育，实施技能型人才贯通培养工程，完善职教园区功能。优先发展民族教育。积极鼓励社会力量办学，实现教育投资多元化。开展校园安全及周边环境清理整顿工作，加强学生品德教育与体能训练。深化国际教育交流，支持本地学校与国内外知名院校合作办学。重视支持高等教育发展，推动构建校企合作、产学研用融合的办学模式。开展全民读书活动，整合各类教育资源，加快发展全民教育、社区教育，建设学习型城市。

加快创建国家健康城市试点。全面推进城市公立医院综合改革，建立健全合理的用人机制和分配机制，关爱医务人员身心健康，增强医务人员职业荣誉感。完善分级诊疗制度，健全药品供应保障机制，减轻群众医疗负担。实施市中心医院北梁分院、六医院门诊综合楼等新建改建项目，均衡配置医疗资源。全面加强专科医院建设，积极发展民办医疗。推动优质医疗卫生资源和医学人才双下沉，强化苏木乡镇卫生院医疗设施配备，推动基层卫生服务机构标准化。加强妇女、儿童、老年人、残疾人等重点人群健康服务，做好公共卫生和重大疾病防控工作。推动蒙中医药事业健康发展。构建计生家庭关爱体系，提升家庭发展能力。完善体育公共服务体系，启动建设冬季奥林匹克体育中心和内蒙古中德足球精英中心。办好足球中乙联赛、市民运动会、环五当召自行车邀请赛等活动，实施全民健身路径和农牧民体育健身工程，引导推动全民健身。

（八）维护和谐稳定，全力推动民主法治建设实现新进步。进一步强化法治思维，加快建设法治包头，不断巩固安定团结的大好局面，大力推进和谐发展。

全面加强法治政府建设。坚决执行市人大及其常委会的决议和决定，坚持重大事项报告制度，密切与政协委员、民主党派、工商联、无党派人士和人民团体的民主协商，自觉接受各方面监督，认真办理人大代表建议和政协提案。健全重大行政决策程序，加强调查研究，完善法律顾问制度，推进科学民主决策。坚持“以公开为常态，不公开为例外”，大力推进政务信息公开，加强政策解读，回应社会关切，让人民群众更好地了解和监督政府工作。

建设更高水平的平安包头。加强社区建设和网格化管理，增强社区服务功能。解决好社区工作人员“进口”与“出口”的问题，提高社区工作者待遇。调动群众参与社区管理积极性，实现政府治理和社会调解、居民自治良性互动。畅通信访渠道，及时妥善处理信访突出问题，解决好群众合理合法的利益诉求。严格落实安全生产责任制和管理制度，坚决遏制重特大事故发生。全面加强应急管理，提高防灾减灾和应急救援能力。积极创建国家食品安全城市，对校园及周边、农贸市场、食品生产小作坊进行专项整治，切实提高食药安全保障能力。推进以信息化为支撑的社会治安防控体系建设，严格执行“三见警”措施，深化提升“四级巡控”机制，大力整治社会治安突出问题。强化禁毒攻坚，深入开展禁毒宣传教育，严厉打击涉毒涉黑违法犯罪。支持驻包军警部队改革发展，加强国防动员和后备力量建设，深化双拥共建，筑牢祖国北疆安全稳定屏障。

努力开创民族团结进步事业新局面。全面贯彻党的民族政策，深化民族团结进步教育和创建活动。深入落实党的宗教工作基本方针，发挥好宗教团体、宗教界人士和信教群众在促进经济社会发展中的积极作用。认真组织好自治区成立70周年庆祝活动，充分展示党的民族区域自治制度的巨大优越性，充分展示内蒙古和包头市的美好前景，进一步增强全市各族干部群众的自豪感和使命感，凝聚起推动改革发展的强大正能量。

各位代表！完成好今年各项目标任务需要付出更加艰辛的努力。我们要在政府系统组织开展“作风转变年”活动，围绕提高执行力和落实力全面加强自身建设。认真落实党风廉政建设主体责任，抓好行政监察和审计监督，坚决整治懒政庸政不作为问题，打造忠诚、干净、担当的公务员队伍。强化政府管理，建立并严格执行各级工作责任制，紧盯不落实的事，问责不落实的人，推动各项目标任务不折不扣落到实处。注重政策研究和工作创新，给改革创新者撑腰鼓劲，不务虚功，但求实效，充分调动各方面的智慧和力量，全力创造一流工作业绩，赢得群众的信赖和支持。

各位代表！美好明天属于包头各族人民，幸福生活要用勤劳智慧来创造。让我们更加紧密地团结在以习近平同志为核心的党中央周围，更加坚定地维护以习近平同志为核心的党中央权威，更加自觉地在思想上政治上行动上同以习近平同志为核心的党中央保持高度一致，更加扎实地把党中央的各项决策部署落到实处，在自治区党委、政府和市委的坚强领导下，汇聚每个人的梦想和追求，激发每个人的激情和创造力，撸起袖子加油干，挺起脊梁振雄风，用人民群众满意的成果向自治区成立70周年献礼，向党的十九大献礼！
